# Hepatic carcinosarcoma with predominant sarcomatous component mimicking intrahepatic cholangiocarcinoma: a case report and literature review

**DOI:** 10.3389/fmed.2026.1805372

**Published:** 2026-04-10

**Authors:** Xuda Ji, Meijia Fu, Fangfeng Liu

**Affiliations:** 1 Department of Hepatobiliary Surgery, Shandong Provincial Hospital Affiliated to Shandong First Medical University, Jinan, Shandong, China; 2 Shandong Provincial Hospital, Shandong University, Jinan, Shandong, China; 3 Shandong First Medical University (Shandong Academy of Medical Sciences), Jinan, Shandong, China

**Keywords:** case report, cholangiocarcinoma mimicry, diagnostic pitfall, hepatic carcinosarcoma (HCS), preoperative misdiagnosis, sarcomatoid differentiation

## Abstract

**Background:**

Hepatic carcinosarcoma (HCS) is an extremely rare primary malignant liver tumor characterized by aggressive biological behavior, early invasion, and poor prognosis. Owing to its non-specific clinical manifestations and imaging features, preoperative diagnosis remains challenging.

**Case report:**

We report a 54-year-old man who presented with persistent fever and unexplained weight loss for 2 weeks. Imaging studies revealed a large space-occupying lesion in the right hepatic lobe, with a preliminary suspicion of cholangiocarcinoma. Liver biopsy findings suggested a sarcomatous lesion. Further computed tomography (CT) evaluation revealed a heterogeneous hypodense mass (11 cm) in the right liver, encircling the right portal vein branch and accompanied by mild bile duct dilatation. Laboratory tests showed normal levels of tumor markers including alpha-fetoprotein (AFP), carcinoembryonic antigen (CEA), carbohydrate antigen 19-9 (CA19-9), and carbohydrate antigen 125 (CA125). The patient subsequently underwent laparoscopic right hepatectomy and cholecystectomy. Postoperative pathology revealed extensive tumor necrosis with residual components consisting of approximately 30% poorly differentiated hepatocellular carcinoma and 70% sarcomatoid tissue, confirming the diagnosis of hepatic carcinosarcoma.

**Conclusion:**

Hepatic carcinosarcoma often presents with insidious and non-specific clinical and radiological features, resulting in frequent misdiagnosis. Complete resection remains the primary treatment modality. This case underscores the importance of integrating imaging, histopathology, and immunohistochemical analysis for confirming this rare tumor.

## 1 Introduction

Hepatic carcinosarcoma (HCS) is a rare malignant tumor characterized by the coexistence and intimate admixture of epithelial and mesenchymal malignant components (1). Primary carcinosarcoma originating in the liver are extremely rare, accounting for less than 1% of HCCs (2, 3). It predominantly affects middle-aged and elderly men and is associated with a poor prognosis, with a reported median postoperative survival of approximately 8 months (4). Due to its low incidence, research on its clinical characteristics, biological behavior, and optimal therapeutic strategies remains limited. The presence of a tumor's biphasic components often complicates diagnosis. Imaging, laboratory tests, and needle biopsies may capture only partial tissue characteristics, leading to misdiagnosis.

Clinically, patients commonly present with non-specific systemic symptoms such as fever, weight loss, and abdominal discomfort (5). Symptoms related to liver dysfunction are often absent until the tumor reaches a considerable size (3). Even when hepatocellular carcinoma is present, serum markers such as AFP often remain within normal ranges, further complicating early detection. Imaging studies often reveal large, heterogeneous liver tumors with variable density or signal intensity, potentially accompanied by areas of necrosis or vascular invasion. However, these features overlap with those of other malignant liver tumors, such as hepatocellular carcinoma or cholangiocarcinoma.

HCS is highly invasive and has a high metastasis rate, often presenting at an advanced or late stage at diagnosis (6). Histopathological examination remains the gold standard for diagnosis. When making a pathological diagnosis, hepatic carcinosarcoma must be differentiated from primary hepatic sarcomatoid carcinoma, as the latter also contains carcinomatous and sarcomatoid components but originates solely from malignant epithelial cell (7, 8). Additionally, immunohistochemistry plays a crucial role in distinguishing between these two components. Glypican-3, HSP70, and CD34 support a hepatocyte origin, while Vimentin and elevated Ki-67 suggest a sarcomatous component and increased proliferative activity.

Herein, we report a case of hepatic carcinosarcoma that radiologically mimicked cholangiocarcinoma and was initially diagnosed as sarcoma on biopsy. Through this case and a brief literature review, we aim to highlight the diagnostic pitfalls and emphasize the importance of integrated clinicopathological evaluation in this rare entity.

## 2 Case presentation

### 2.1 Medical history and clinical symptoms

A 54-year-old male patient presented with persistent recurrent fever for 2 weeks (body temperature approximately 38 °C), without abdominal pain, nausea, vomiting, or other gastrointestinal symptoms. The patient also experienced weight loss (approximately 3.5 kg over 2 months). He had no history of hepatitis virus infection and denied smoking or alcohol consumption. His past medical history was otherwise unremarkable.

### 2.2 Imaging studies

#### 2.2.1 Magnetic resonance imaging (MRI) findings

**Figure 1:**
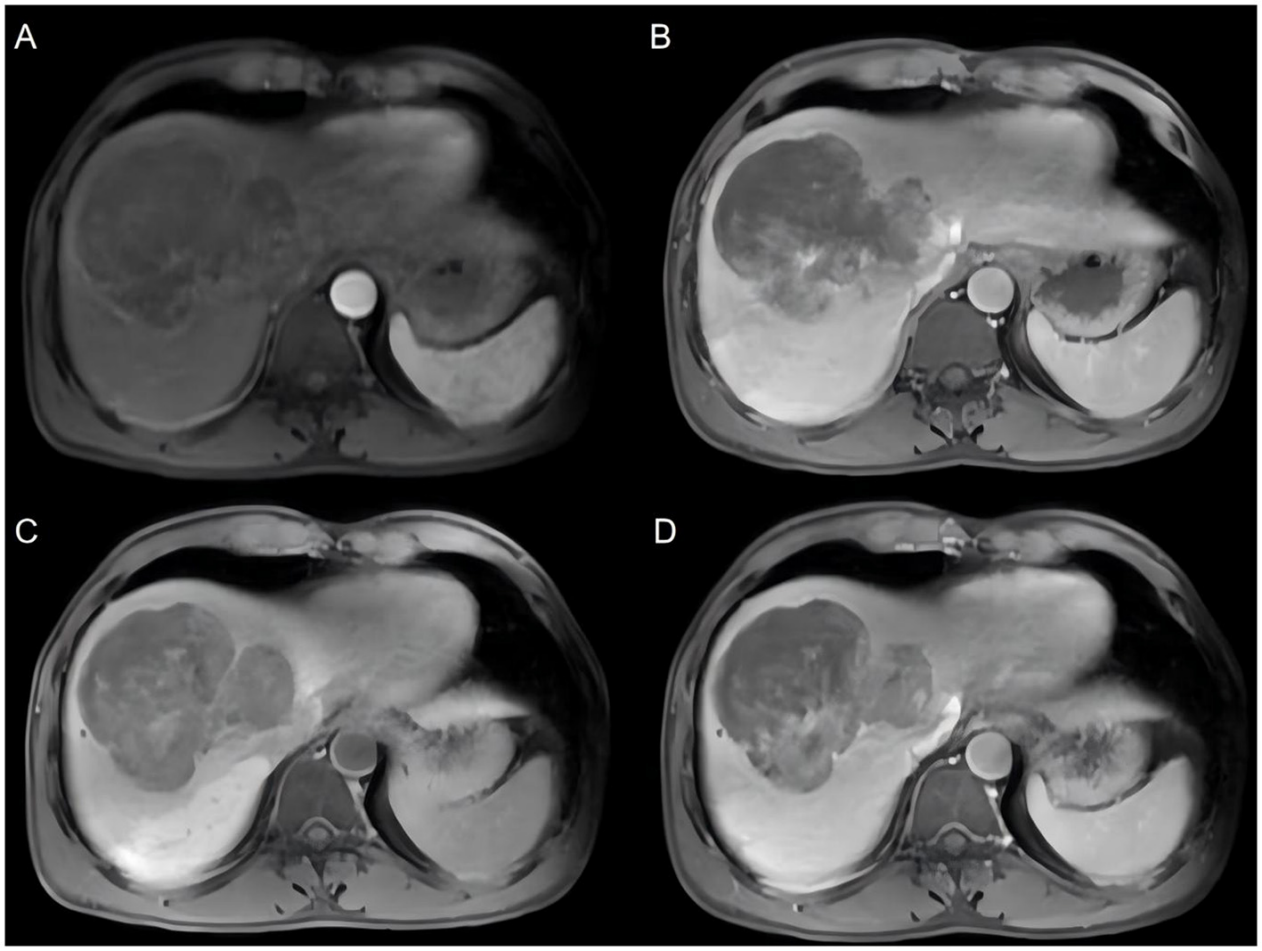
Magnetic resonance imaging (MRI) findings of the hepatic lesion. **(A)** Axial T1-weighted image demonstrates an iso- to slightly hypointense mass in the right hepatic lobe. **(B)** T2-weighted image shows a heterogeneous lesion with relatively hyperintense signal intensity. **(C–D)** Dynamic contrast-enhanced images reveal mild and heterogeneous enhancement of the tumor, with large non-enhancing areas consistent with extensive intratumoral necrosis. Overall, the lesion exhibits hypovascular imaging characteristics, which may mimic intrahepatic cholangiocarcinoma.

Conventional ultrasonography and contrast-enhanced ultrasonography (CEUS) were not performed preoperatively in this patient. Upper abdominal magnetic resonance imaging (MRI) with plain scan and dynamic contrast-enhanced imaging: extensive patchy abnormal signal intensity is observed within the liver parenchyma. On T1-weighted images (T1WI), the lesion exhibits isointense to slightly hyperintense signal intensity; on T2-weighted images (T2WI), it shows isointense to slightly hypointense signal intensity (Figure 1). On T1WI, the lesion demonstrates slightly hypointense signal intensity in the same phase and no significant reduction in signal intensity in the opposite phase. On diffusion-weighted imaging (DWI), the lesion appears hyperintense, with markedly reduced ADC values. The lesion shows mild enhancement in the equilibrium phase and hepatobiliary phase, with enhancement below that of the liver parenchyma. The size of the lesion is approximately 10.9 × 9.0 × 10.1 cm. The right branch of the hepatic vein and the right branch of the portal vein are partially obscured. There is localized dilatation of the intrahepatic bile ducts. An additional small, round, long T2 signal lesion is noted in the liver, showing no enhancement on contrast-enhanced imaging. The gallbladder is not enlarged, the wall is not thickened, and no abnormal signals are seen within the lumen. The spleen is enlarged, with no abnormalities in its parenchymal signal. No enlarged lymph nodes are observed in the abdominal cavity or retroperitoneal space.

#### 2.2.2 Upper abdominal CT scan with contrast enhancement shows

**Figure 2:**
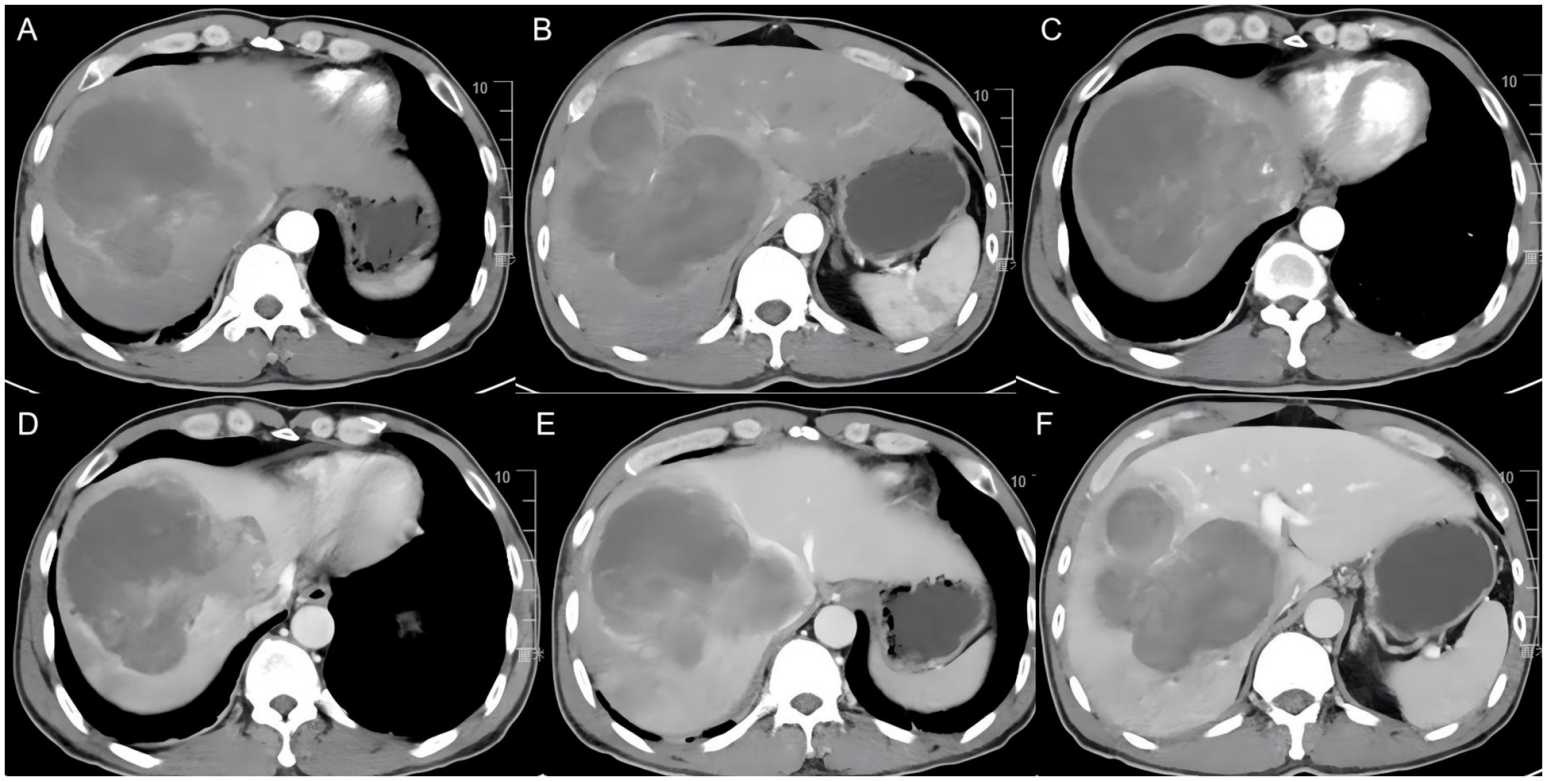
Contrast-enhanced computed tomography (CT) images of the liver. **(A–C)** Axial CT images at different levels demonstrate a large heterogeneous hypodense mass occupying the right hepatic lobe. **(D–F)** Contrast-enhanced phases show mild and heterogeneous enhancement of the lesion, which remains lower than the surrounding liver parenchyma. The tumor exhibits irregular internal architecture with extensive low-density areas suggestive of necrosis and exerts mass effect on adjacent hepatic structures, imaging features that are highly suggestive of a hypovascular malignant hepatic tumor.

**Figure 3:**
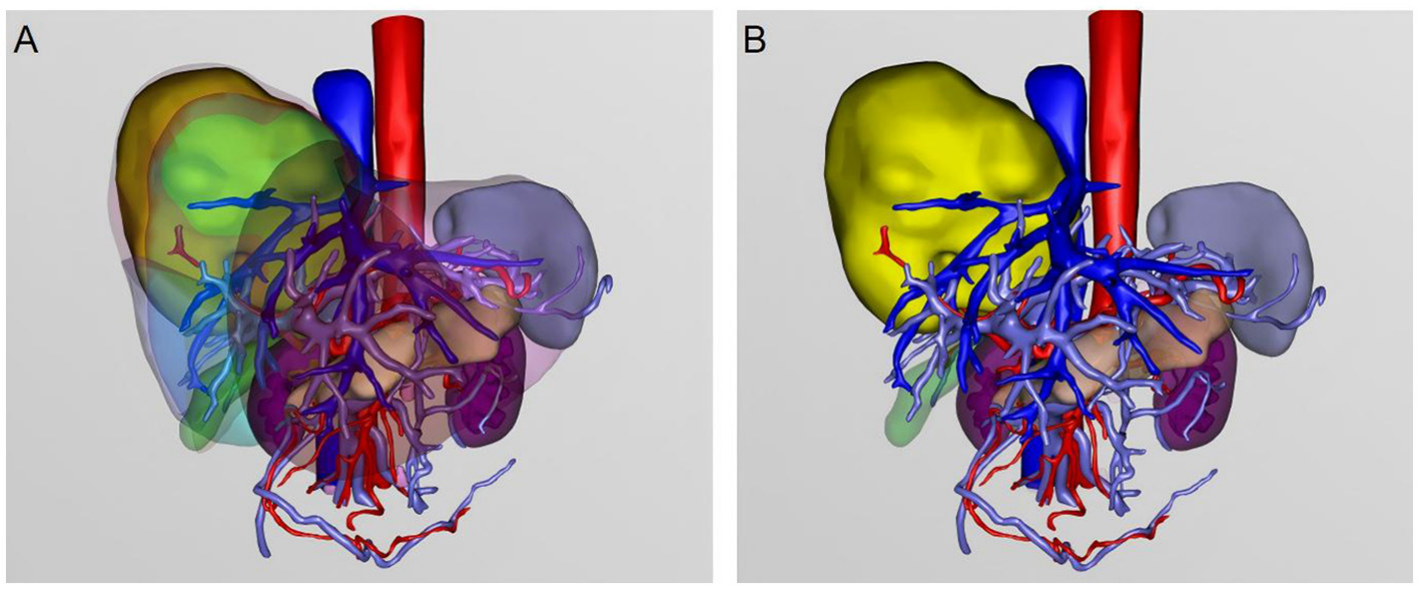
Three-dimensional (3D) reconstruction of the liver, tumor, and intrahepatic vascular structures based on contrast-enhanced imaging. **(A)** Semi-transparent rendering of the liver demonstrates the spatial relationship between the hepatic tumor and surrounding vascular anatomy. **(B)** Isolated visualization of the tumor and major intrahepatic vessels highlights tumor involvement and encasement of the portal vein branches. The hepatic artery is shown in red, the portal vein in blue, and the hepatic veins in light purple. The tumor is primarily located in the right hepatic lobe and exhibits close anatomical association with the right portal vein branch.

An abnormally enhanced lesion in the right hepatic lobe consistent with a neoplastic lesion, suspected to be malignant. A roughly circular, slightly hypodense lesion in the right hepatic lobe, measuring approximately 11.2 × 11.1 cm, with heterogeneous internal density and visible cord-like structures. The lesion demonstrates heterogeneous abnormal enhancement on contrast-enhanced scans, with a degree of enhancement lower than the liver parenchyma (Figure 2). Peripheral bile ducts show slight dilatation. The lesion encircles the right branch of the portal vein, causing compression and narrowing of the middle and right hepatic veins. A cholangiocarcinoma is highly suspected. Three-dimensional (3D) reconstruction further delineated the spatial relationship between the tumor and intrahepatic vessels, confirming encasement of the right portal vein branch (Figure 3).

### 2.3 Pathological examination

#### 2.3.1 Preoperative biopsy

Histopathological examination of the liver mass biopsy revealed a malignant tumor with features suggestive of sarcoma. Immunohistochemistry results: CD34 (vascular +), CD10 (scattered +), HSP70 (+), Vimentin (+), P63 (focal +), CK (–), CK5/6 (–), CK7 (–), CK19 (–), Glypican-3 (–), HepPar-1 (–), AFP (–), Ki-67 (index 35%).

### 2.4 Laboratory tests

Laboratory tests upon admission showed mild anemia on complete blood count (Hb 106 g/L), with white blood cell and platelet counts essentially normal. Liver function tests revealed mildly elevated transaminases (AST 51 U/L, ALT 61 U/L), markedly increased cholestasis-related markers (GGT 274 U/L, ALP 332 U/L), and decreased albumin (31.8 g/L). Bilirubin levels were mildly elevated (DBIL 16.3 μmol/L). Tumor markers were within normal ranges: AFP (1.20 ng/mL), CEA (0.95 ng/mL), CA125 (22.20 U/mL), and CA19-9 (11.10 U/mL). However, protein induced by vitamin K absence or antagonist-II (PIVKA-II) was elevated to 136 mAU/mL.

### 2.5 Treatment and outcomes

Preoperatively, the patient was assessed as having a resectable hepatic mass, and surgical resection was planned. Laparoscopic exploration revealed a small amount of ascites in the abdominal cavity. The gallbladder appeared normal in size and morphology, and no abnormalities were observed in the pelvis or greater omentum. A friable tumor measuring approximately 11 × 11 cm was identified in the right lobe of the liver, while the remaining liver parenchyma exhibited normal coloration and soft consistency. Based on preoperative imaging confirming a right hepatic tumor, a laparoscopic right hepatectomy with cholecystectomy was performed. The right hepatic lobe containing the tumor and the gallbladder were completely resected en bloc. Intraoperative rapid pathological examination demonstrated extensive tumor necrosis with only sparse viable tumor cells at the resection margins. The morphology was consistent with malignancy; however, due to the minimal viable tumor component, the epithelial or mesenchymal origin could not be determined, and further diagnosis was deferred pending paraffin-embedded sections and immunohistochemical analysis. The posterior right hepatic hilar bile duct stump was sutured, and the hepatic resection margin was carefully inspected, confirming no active bleeding or bile leakage. Two drainage tubes were placed at the resection site and exteriorized through the abdominal wall. The estimated intraoperative blood loss was approximately 1,000 ml, and the patient received transfusions of 4 units of packed red blood cells and 400 ml of fresh frozen plasma. The postoperative course was uneventful, and the patient was discharged 6 days after removal of the abdominal drainage tubes.

### 2.6 Final pathological findings

**Figure 4:**
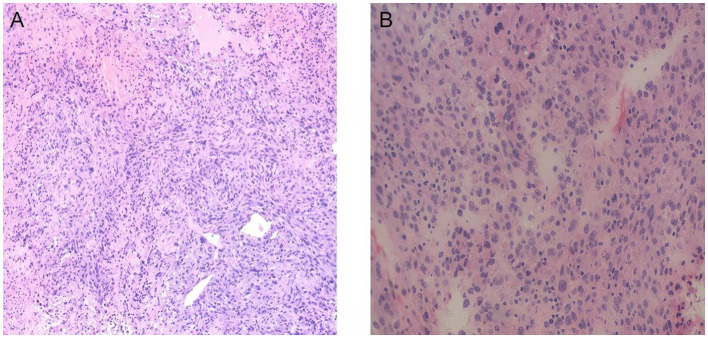
Histopathological findings of the hepatic tumor on hematoxylin and eosin (H&E) staining. **(A)** Low-power magnification demonstrates a densely cellular tumor composed predominantly of spindle-shaped cells arranged in interlacing fascicles. **(B)** High-power magnification reveals marked cellular atypia with pleomorphic nuclei and an increased nuclear-to-cytoplasmic ratio. No definitive epithelial differentiation is identified in these fields, supporting sarcomatous differentiation and constituting the sarcomatous component of hepatic carcinosarcoma.

The final pathological findings are shown in Figure 4. Extensive necrosis of the hepatic lesion with scattered viable tumor cells at the margins. The viable tumor cell component is minimal, and the histological features are consistent with sarcoma. The resected tumor measures 11 × 8 cm and exhibits a friable consistency. Approximately 30% of the specimen consists of hepatocellular carcinoma (grade 3); the remaining 70% demonstrates sarcomatous morphology, consistent with carcinosarcoma. The surrounding non-neoplastic liver parenchyma showed mild chronic inflammatory infiltration without evidence of cirrhosis or significant fibrosis. Chronic cholecystitis is also noted.

### 2.7 Immunohistochemical analysis

**Hepatocellular carcinoma region:** CD34(+), Glypican-3(+), HSP70(+), CD68(+); **Sarcoma area:** CD34 (+), Vimentin (+), Ki-67 (+30%), CK (AE1/AE3) (–), S100 (–), HMB45 (–), SMA (–), SS18 (–), TTF1 (–), CD117 (–), Dog-1 (–).

## 3 Discussion

Hepatic carcinosarcoma (HCS) is an extremely rare and highly aggressive primary malignant tumor of the liver, characterized by the coexistence and intermingling of carcinomatous and sarcomatous components within the same tumor (9, 10). Previous studies have reported that HCS accounts for less than 1% of all hepatocellular carcinomas, and most cases are diagnosed at an advanced stage, with a very poor prognosis and a median survival time of approximately 8 months (2, 4). Due to the lack of specific clinical manifestations, imaging features, and serological markers, early diagnosis of HCS remains a major challenge in clinical practice.

In the present case, the patient initially presented with persistent recurrent fever and weight loss, without typical symptoms related to liver cancer. Serum tumor markers, including AFP, CEA, and CA19-9, were within normal ranges, while only abnormal prothrombin (PIVKA-II) was elevated (11, 12). Previous studies have shown that even when a clear hepatocellular carcinoma component is present, AFP levels in patients with HCS may remain normal, thereby masking the malignant nature of the disease and delaying diagnosis (13). This case suggests that in patients with unexplained fever and weight loss, the possibility of hepatic malignancy should still be considered, even when routine tumor markers are negative (14).

From an imaging perspective, the tumor appeared as a giant mass in the right hepatic lobe with markedly heterogeneous signal intensity and density. Diffusion-weighted imaging demonstrated high signal intensity with reduced apparent diffusion coefficient values, indicating high cellular density and malignant characteristics (15). Contrast-enhanced imaging showed mild and heterogeneous enhancement, which was overall lower than that of the surrounding liver parenchyma. However, these imaging features lack specificity and overlap considerably with those of other common hepatic malignancies, such as cholangiocarcinoma, poorly differentiated hepatocellular carcinoma, and even intrahepatic metastatic tumors, making accurate preoperative diagnosis based on imaging extremely difficult (11). Notably, despite its large size, the tumor did not exhibit significant arterial-phase enhancement, which is inconsistent with typical hypervascular hepatocellular carcinoma. As a result, it was more likely to be misdiagnosed as a biliary-origin tumor or another hypovascular malignancy, further increasing the risk of misdiagnosis. Based on postoperative pathological findings, these imaging characteristics may be closely related to extensive intratumoral necrosis and a high proportion of sarcomatous components, leading to heterogeneous blood supply. This provides an imaging-based explanation for the difficulty in accurately identifying the tumor nature preoperatively in this case. Although CEUS was not performed in the present case, previous studies have reported that CEUS findings in hepatic carcinosarcoma may demonstrate heterogeneous peripheral enhancement during the arterial phase, with a central non-enhancing area suggestive of tumor necrosis, followed by rapid washout in the portal venous phase. These imaging features may provide additional diagnostic clues for malignant hepatic tumors (10, 16). Therefore, multimodal imaging evaluation may help improve the preoperative diagnostic accuracy for this rare tumor. In retrospect, the absence of CEUS represented a limitation of the preoperative imaging work-up in this case.

Postoperative histopathological examination remains the cornerstone for the definitive diagnosis of hepatic carcinosarcoma (11). In this case, preoperative needle biopsy suggested only a sarcomatous lesion and failed to identify the carcinomatous component, reflecting a typical limitation of biopsy in the diagnosis of HCS (13). This limitation is mainly attributed to the uneven distribution of tumor components and extensive necrosis, which makes single-point sampling prone to missing carcinomatous areas. Examination of the surgical specimen ultimately revealed that the tumor consisted of approximately 30% poorly differentiated hepatocellular carcinoma components and 70% sarcomatoid components, meeting the diagnostic criteria for hepatic carcinosarcoma (12). Immunohistochemical analysis further confirmed the biphasic differentiation pattern. In the differential diagnosis, primary hepatic sarcomatoid carcinoma should be carefully excluded, as its sarcomatoid components usually retain expression of epithelial markers (4, 13). In the present case, the sarcomatous areas were completely negative for epithelial markers such as CK (AE1/AE3), and the presence of a definite hepatocellular carcinoma component further supported the diagnosis of hepatic carcinosarcoma.

The pathogenesis of hepatic carcinosarcoma (HCS) remains controversial. Two major hypotheses have been proposed: the collision theory and the combination theory. The collision theory, also known as the biclonal theory, suggests that epithelial carcinoma and mesenchymal sarcoma arise independently and subsequently collide and merge within the liver. In contrast, the combination theory, also referred to as the monoclonal theory, proposes that both tumor components originate from the same tumor stem cell or the same cellular clone and subsequently undergo divergent differentiation during tumor progression, resulting in the formation of sarcomatous components (1, 8). In the present case, the carcinomatous and sarcomatous components were closely intermingled histologically without clear boundaries, which more strongly favors the transformation theory (monoclonal origin) rather than the collision theory (3, 17).

Although case-specific genomic testing was not performed in the present patient, recent molecular studies have provided new insights into the genetic alterations of HCS. Microsatellite and genomic analyses have demonstrated that the carcinomatous and sarcomatous components of the tumor may share identical genetic mutations, further supporting the monoclonal origin hypothesis (1). Reported genetic alterations include TP53, KRAS, and TERT promoter mutations (18). In addition, dysregulation of signaling pathways associated with epithelial–mesenchymal transition (EMT) has been implicated in sarcomatoid transformation and increased tumor aggressiveness (19). Further investigation of these molecular mechanisms may not only improve the understanding of HCS pathogenesis but also provide potential targets for future molecular-targeted therapies. The lack of molecular profiling in this case should therefore be regarded as a limitation of the present report.

Hepatic carcinosarcoma generally carries a poor prognosis, mainly due to its highly aggressive biological behavior (20). Previous studies have suggested that several factors may influence patient outcomes, including large tumor size, vascular invasion, extensive necrosis, and a high proportion of sarcomatous components. In addition, postoperative recurrence and distant metastasis are relatively common in this disease (12). In the present case, the tumor exceeded 10 cm in diameter and contained a high proportion of sarcomatoid components, both of which indicate potentially aggressive tumor behavior. Moreover, preoperative imaging demonstrated encasement of the right portal vein branch and compression of the middle and right hepatic veins, suggesting locally advanced disease with possible adverse prognostic implications. The extensive tumor necrosis identified on pathological examination may also reflect rapid tumor progression and marked intratumoral heterogeneity, both of which are consistent with aggressive biological behavior. However, the patient experienced rapid resolution of fever and gradual recovery of body weight following surgery, suggesting that aggressive surgical resection may still provide clinical benefit in selected patients (20). Nevertheless, given the recognized risk of recurrence and distant metastasis in HCS, close postoperative surveillance remains essential.

At present, there are no established or unified treatment guidelines for hepatic carcinosarcoma (21). When tumor resectability is confirmed, surgical resection remains the preferred treatment option. Previous studies have shown that even after complete resection, HCS carries a high risk of recurrence and metastasis, and the median survival time is significantly shorter than that of more common hepatic malignancies (4, 20). However, in this case, the patient experienced marked relief of fever and weight gain after surgery, suggesting that aggressive surgical intervention may still provide short-term clinical benefit in selected patients. Given the limited evidence supporting postoperative adjuvant therapy, long-term follow-up and individualized management under a multidisciplinary approach are particularly important. For this reason, regular postoperative surveillance should be emphasized in order to detect early recurrence or distant metastasis as promptly as possible.

This case report has several limitations. First, contrast-enhanced ultrasonography (CEUS) was not performed, which restricted multimodal imaging comparison before surgery. Second, molecular profiling was not available in this patient, and therefore case-specific genetic alterations could not be characterized. Nevertheless, the combination of imaging findings, histopathological features, and immunohistochemical results still provides useful evidence for the diagnosis of hepatic carcinosarcoma and for discussing its possible histogenesis, biological behavior, and clinical management.

## 4 Conclusion

Hepatic carcinosarcoma is a rare and highly aggressive primary malignant tumor of the liver. Owing to the non-specific clinical manifestations, imaging findings, and serum tumor markers, accurate preoperative diagnosis remains difficult and is often associated with misdiagnosis. Even in cases with normal AFP levels and imaging features suggestive of cholangiocarcinoma, the possibility of a hepatocellular carcinoma component should still be considered. Given the marked intratumoral heterogeneity and extensive necrosis, preoperative needle biopsy has inherent diagnostic limitations. Histopathological and immunohistochemical examination of the complete resection specimen remains the gold standard for definitive diagnosis. At present, there is no unified treatment strategy for hepatic carcinosarcoma, and complete surgical resection remains the preferred therapeutic approach, which may provide survival benefit for selected patients. Future incorporation of multimodal imaging, including CEUS, together with molecular profiling may further improve diagnostic accuracy and deepen understanding of the biological behavior of this rare tumor.

## Data Availability

The original contributions presented in the study are included in the article/supplementary material, further inquiries can be directed to the corresponding author.
